# Flavonoids activate pregnane × receptor-mediated *CYP3A4 *gene expression by inhibiting cyclin-dependent kinases in HepG2 liver carcinoma cells

**DOI:** 10.1186/1471-2091-11-23

**Published:** 2010-06-16

**Authors:** Hanqing Dong, Wenwei Lin, Jing Wu, Taosheng Chen

**Affiliations:** 1 Department of Chemical Biology and Therapeutics, St. Jude Children's Research Hospital, 262 Danny Thomas Place, Memphis, TN 38105, USA; 2 Albert Einstein College of Medicine, 1300 Morris Park Ave., Ullmann 609, Bronx, NY 10461, USA

## Abstract

**Background:**

The expression of the drug-metabolizing enzyme cytochrome P450 3A4 (CYP3A4) is regulated by the pregnane × receptor (PXR), which is modulated by numerous signaling pathways, including the cyclin-dependent kinase (Cdk) pathway. Flavonoids, commonly consumed by humans as dietary constituents, have been shown to modulate various signaling pathways (e.g., inhibiting Cdks). Flavonoids have also been shown to induce *CYPs *expression, but the underlying mechanism of action is unknown. Here, we report the mechanism responsible for flavonoid-mediated PXR activation and *CYP *expression.

**Results:**

In a cell-based screen designed to identify compounds that activate PXR-mediated *CYP3A4 *gene expression in HepG2 human carcinoma cells, we identified several flavonoids, such as luteolin and apigenin, as PXR activators. The flavonoids did not directly bind to PXR, suggesting that an alternative mechanism may be responsible for flavonoid-mediated PXR activation. Consistent with the Cdk5-inhibitory effect of flavonoids, Cdk5 and p35 (a non-cyclin regulatory subunit required to activate Cdk5) were expressed in HepG2. The activation of Cdk5 attenuated PXR-mediated *CYP3A4 *expression whereas its downregulation enhanced it. The Cdk5-mediated downregulation of *CYP3A4 *promoter activity was restored by flavonoids, suggesting that flavonoids activate PXR by inactivating Cdk5. *In vitro *kinase assays showed that Cdk5 directly phosphorylates PXR. The Cdk kinase profiling assay showed that apigenin inhibits multiple Cdks, suggesting that several Cdks may be involved in activation of PXR by flavonoids.

**Conclusions:**

Our results for the first time link the stimulatory effect of flavonoids on *CYP *expression to their inhibitory effect on Cdks, through a PXR-mediated mechanism. These results may have important implications on the pharmacokinetics of drugs co-administered with herbal remedy and herbal-drug interactions.

## Background

The pregnane × receptor (PXR) is a key xenobiotic receptor that regulates the metabolism and excretion of xenobiotics and endobiotics by regulating the expression of drug-metabolizing enzymes and drug transporters [1,2]. Expression of PXR target gene is regulated by binding of PXR to its promoter region, such as that of cytochrome P450 3A4 (CYP3A4), a key enzyme that catalyzes the metabolism of more than 50% of all clinically prescribed drugs [3]. Changes in the expression of *CYP3A4 *affect drug metabolism and alter the therapeutic and toxicologic responses to drugs, which may in turn lead to adverse drug interactions.

The activity of PXR is regulated not only by direct ligand binding [4] but also by various cell signaling pathways [5], such as those mediated by protein kinase C (PKC) [6], protein kinase A (PKA) [7,8], cyclin-dependent kinase 2 (Cdk2) [9], 70kDa form of ribosomal protein S6 kinase (p70 S6K) [10], forkhead in rhabdomyosarcoma (FKHR) [11], and nuclear factor κB (NF-κB) [12-14].

Flavonoids - secondary metabolites found ubiquitously in plants - are the most common group of polyphenolic compounds consumed by humans as dietary constituents [15]. Thousands of naturally occurring flavonoids, such as flavones and isoflavones, have been characterized [16]. Flavonoids have been reported to have anti-allergic, anti-inflammatory, anti-microbial and anti-cancer activities [17,18]. The widespread use of flavonoids, coupled with their potentially beneficial effects, has triggered studies on the mechanism by which they modulate signaling pathways.

Natural flavonoids have been shown to inhibit Cdk1, Cdk2 [19], and Cdk5 [20]. Most Cdks, including Cdk1 and Cdk2, are involved in cell cycle regulation and require the binding of cyclins for their activation. However, the activation of Cdk5 requires one of the two non-cyclin regulatory subunits p35 or p39, which have 57% amino acid homology. p35 can be converted in a Ca^2+^-dependent manner to p25, a highly active and stable proteolytic product [21,22]. The protease calpain catalyzes the cleavage of p35, and this reaction can be effectively inhibited by specific inhibitors of calpain such as calpeptin [23,24]. Cdk5 is not involved in cell cycle progression, and is expressed in all tissues, but its levels of expression and activity are highest in the nervous system [21,25]. The expressions of p35 and p39 are also highest in the nervous system. Although Cdk5 has been mainly implicated in early development of the central nervous system (CNS) and maintenance of neuronal architecture [21,26], the expression and regulatory activity of Cdk5/p35 have also been reported in several non-CNS tissues such as lens epithelia [27], muscle tissues [28], hepatoma cells [25], adipose tissues [29], and male reproductive system [30].

The widespread use of flavonoids has triggered studies to investigate their effects on drug metabolism and herbal-drug interactions. Recently, flavonoids have been shown to induce *CYP *expression through PXR [31-36], but the mechanism of flavonoids-mediated PXR activation and CYP induction remain unknown.

Because the function of PXR can be modulated by cellular signaling pathways, we used a cell-based screening approach in this study to identify compounds with known bioactivities that activate PXR-mediated gene expression. By screening a library of known bioactive compounds, we identified a series of flavonoids that are PXR activators. Since these flavonoids did not directly bind to PXR, and flavonoids might inhibit Cdk5, we studied the effect of flavonoids on the activity of Cdk5/p35 and the regulation of PXR by Cdk5 in order to determine the possible role of flavonoids in regulating PXR-mediated gene expression of *CYP3A4*.

## Results

### Flavonoids activate PXR-mediated CYP3A4 gene expression

**Figure 1:**
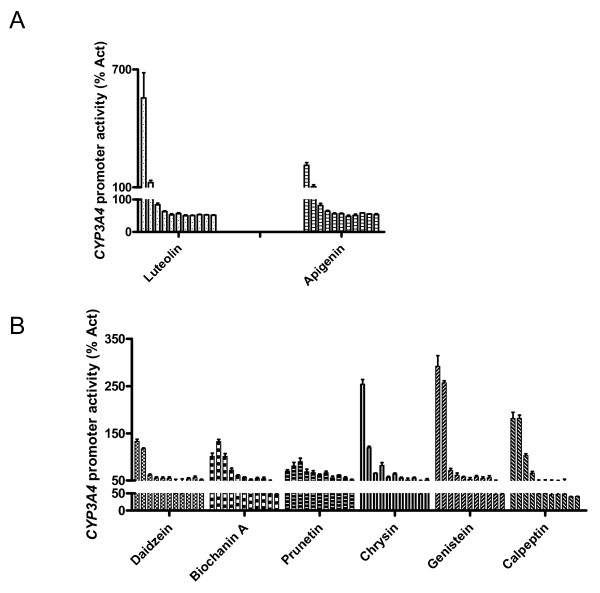
**Flavonoids activate PXR-mediated *CYP3A4 *gene expression**. HepG2 cells (2 × 10^4^/well) stably transfected with PXR and *CYP3A4*-luc [9] were seeded in 384-well plates and treated with indicated compounds for 24 h before the luciferase assay. *CYP3A4 *promoter activity induced by compound was expressed as a percentage of activation (% Act) by normalizing with luciferase activity from 2 μM rifampicin. Compounds were tested at 11 concentrations starting at 40 μM (leftmost for each compound), 1:1.5 (A) or 1:3 (B) serial dilutions. In (A), the concentrations for each compound tested were 40, 26.667, 17.778, 11.852, 7.901, 5.267, 3.512, 2.341, 1.561, 1.040 and 0.694 μM (from left to right). In (B), the concentrations for each compound tested were 40, 13.333, 4.444, 1.481, 0.494, 0.165, 0.055, 0.018, 0.006, 0.002 and 0.0007 μM (from left to right). Compounds were tested in quadruplicate. The bars denote the standard deviation.

By screening a library of 3200 compounds with known bioactivity in the human carcinoma cell line HepG2 stably transfected with PXR and *CYP3A4*-luc, which was previously used to detect the activation PXR [9], we identified a series of flavonoids as potent activators of PXR-mediated *CYP3A4 *promoter activation (Fig. 1). These flavonoids included flavones luteolin, apigenin (Fig. 1A), and chrysin (Fig. 1B) and isoflavones daidzein, biochanin A, prunetin, and genistein (Fig. 1B). Rifampicin, a human PXR agonist, was used as a control in this assay, and had an EC_50 _of 1.3 μM. Compared with the activation of PXR by rifampicin at 2 μM (a concentration at which PXR is activated without causing cytotoxicity), some flavonoids were more potent at activating PXR at high concentrations. For example, luteolin at 40 μM was 7 times more effective than 2 μM rifampicin in activating PXR. Under the same assay conditions and compound treatment time (24 h) as the PXR transactivation assay described above, no significant cytotoxicity was detected for all flavonoids tested (data not shown).

**Figure 2:**
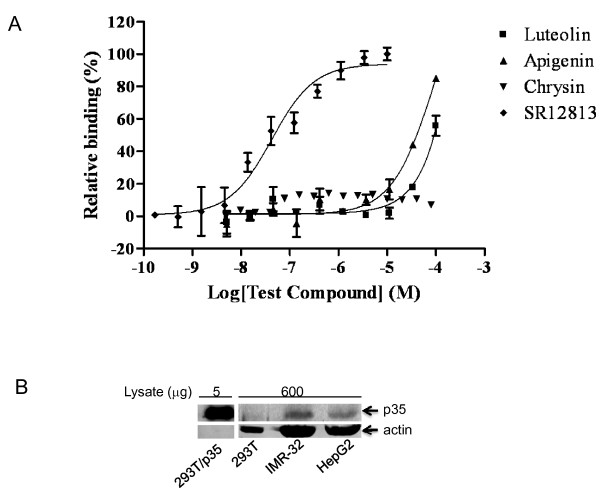
**Chrysin, apigenin, and luteolin are not potent PXR binders; p35 is expressed in HepG2**. (A) Chrysin, apigenin, and luteolin are not potent PXR binders. SR12813, a potent human PXR agonist, was used as a positive control. Relative binding (%) was determined as described in Methods. Compounds were tested in triplicate. The bars denote the standard deviation. (B) p35 is expressed in HepG2 (lane 4). HEK 293T (293T) cells transfected with a p35 expression construct (293T/p35) (8 × 10^5 ^cells in 6-well plates transfected with 1 μg of pCMV-mycP35 using Fugene 6) was used as a positive control for p35 expression (lane 1). 293T was used as a negative control (lane 2). IMR-32, a neuroblastoma cell line that expresses p35 endogenously, was used as an additional positive control (lane 3). Total amount of lysate (μg) used is indicated above the samples. Since only 5 μg of total protein was used in lane 1, no actin band was detected.

To determine whether the flavonoids activate PXR by directly binding to it, we tested 3 flavonoids (chrysin, luteolin, and apigenin) in a PXR-binding assay. Although the potent PXR agonist SR-12813 bound strongly (Fig. 2A; IC_50 _= 42 nM) to PXR, chrysin did not bind to PXR at all concentrations tested (Fig. 2A). Luteolin and apigenin did not bind to PXR at or below 10 μM. However, below 10 μM, they strongly activated PXR (more than 50% of the activity from 2 μM of rifampicin) (Fig. 1A). These data suggest that mechanisms other than direct PXR binding might be responsible for chrysin-, luteolin-and apigenin-mediated PXR activation.

### Activation of Cdk5/p35 attenuates PXR-mediated gene expression

Flavonoids have been shown to inhibit protein kinases, including Cdks [19,20]. Flavonoids may regulate PXR by inhibiting Cdk2, as Cdk2 has been shown to negatively regulate PXR [9]. However, because flavonoids can inhibit Cdk5 [20] and Cdk5/p35 signaling (which is not regulated by cell cycling) is active in hepatoma [25], we tested whether inhibition of Cdk5 by flavonoids is responsible for the flavonoids-mediated activation of PXR.

Since the activity of Cdk5 requires p35 as a critical regulatory subunit, we determined whether p35 is expressed in HepG2 cells, in which flavonoid-mediated activation of PXR was first discovered. We found that p35 was expressed in HepG2 cells at levels comparable to those in IMR-32 (Fig. 2B), a neuronal cell line that expresses p35 and has been used as a positive control for p35 expression [25].

**Figure 3:**
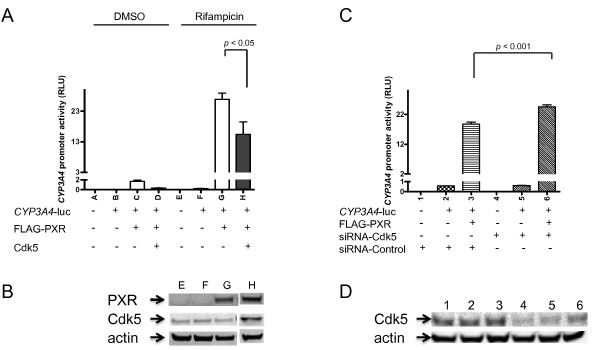
**Cdk5 activation attenuates PXR-mediated gene expression**. (A) Overexpression of Cdk5 reduces PXR-mediated *CYP3A4 *promoter activity. HepG2 was transiently co-transfected with indicated plasmids as well as CMV-*renilla *luciferase plasmid (as a transfection control). In cases wherein Cdk5, FLAG-PXR, or CYP3A4-luc constructs were not used, a pcDNA3 vector was used instead. Equal amount of each plasmid (for a total of 1 μg combined) was used to transfect 8 × 10^5 ^cells seeded in 6-well plates. Cells were treated with 5 μM rifampicin or DMSO for 24 h after transfection before Dual-glo luciferase assay. *CYP3A4 *promoter activity (expressed as relative luciferase unit, or RLU) was normalized by using activity of the CMV-*renilla*. The values represent the average of 8 independent experiments, with the standard deviation denoted as bars. The significance of the difference between datasets was determined by using the Student's *t *test. (B) Expression of PXR and Cdk5. Transfections were performed as described in (A). Western blotting shown was from a representative experiment. (C) Downregulation of Cdk5 enhances the activity of PXR. HepG2 was transfected with siRNA specific for Cdk5 (siRNA-Cdk5) or control siRNA (siRNA-Control) in addition to indicated plasmids and CMV-*renilla *as described in Methods. Cells were treated with 5 μM rifampicin. *CYP3A4 *promoter activity was expressed as RLU as described in (A). The values represent the average of 6 independent experiments. The efficiency of Cdk5 knockdown was verified in (D).

**Figure 4:**
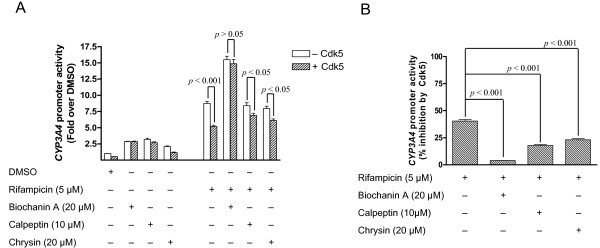
**Flavonoids and calpeptin decrease the inhibitory effect of Cdk5 on PXR-mediated *CYP3A4 *promoter activity**. (A) HepG2 was transiently co-transfected with FLAG-PXR, *CYP3A4*-luc, CMV-*renilla *luciferase (as a transfection control), and either Cdk5 (+ Cdk5) or pcDNA3 vector (-Cdk5) plasmids at a ratio of 1:15:1:3, respectively. A total of 20 μg of combined plasmids was used to transfect 5 × 10^6 ^cells seeded in a T75 flask 16 h before transfection. Transfected cells were seeded into white 96-well plates (at 10,000 cells/well) 24 h later, and treated with DMSO or compounds as indicated for 24 h before Dual-glo luciferase assay. *CYP3A4 *promoter activity was normalized by using activity of the CMV-*renilla*, and expressed as fold induction over control (DMSO; without Cdk5 transfection). The values represent the average of 8 independent experiments, with the standard deviation denoted as bars. The significance of the difference between datasets was determined by using the Student's *t *test. (B) Using fold induction over control as described in (A), the Cdk5-mediated inhibition of *CYP3A4 *promoter activity was expressed a percentage of inhibition by Cdk5 [% inhibition = 100% × (signal without Cdk5 - signal with Cdk5)/signal without Cdk5)].

Next, we determined the functional correlation between the activities of Cdk5 and PXR. Overexpression of Cdk5 led to attenuation of both basal (DMSO control) and rifampicin-induced activation of PXR (Fig. 3A). Expression levels of PXR were not affected by overexpression of Cdk5 (Fig. 3B), confirming that the attenuation of PXR activity is because of the inhibitory effect of Cdk5 on PXR and not because of a decrease in expression level of PXR. The inhibitory effect of Cdk5 on PXR was further confirmed by the increase in PXR activity on siRNA-mediated downregulation of Cdk5 (Fig. 3C). Reduced expression of Cdk5 in response to siRNA treatment was verified (Fig. 3D). In addition, we showed that flavonoids significantly decreased the inhibitory effect of Cdk5 on *CYP3A4 *promoter activity induced by rifampicin (Fig. 4). In the absence of flavonoids, Cdk5 inhibited *CYP3A4 *promoter activity by 40%. The inhibitory effect of Cdk5 was decreased to 4% and 23% by 20 μM of biochanin A and 20 μM of chrysin, respectively (Fig. 4B). These results suggest that flavonoids may inhibit Cdk5 and restore the Cdk5-mediated downregulation of *CYP3A4 *promoter activity.

To further validate the role of Cdk5 in regulating PXR function, we examined the effect of calpeptin on PXR function. Calpeptin has been shown to block the conversion of p35 to the highly active p25, thereby reducing the activity of Cdk5 [23,24]. Therefore we anticipated that the calpeptin-mediated inhibition of Cdk5 would lead to activation of PXR, and calpeptin may restore the Cdk5-mediated downregulation of *CYP3A4 *promoter activity. Indeed, we found that calpeptin induced PXR activity (e.g., 4.4 μM of calpeptin is as potent as 2 μM of rifampicin) (Fig. 1B), and significantly (*p *< 0.001) decreased the inhibitory effect of Cdk5 on the activity of *CYP3A4 *promoter (Fig. 4B). Taken together, these data indicate that Cdk5 negatively regulates PXR activity, and that inhibition of Cdk5 is at least partially responsible for flavonoids-induced activation of PXR.

### Cdk5 phosphorylates PXR

**Figure 5:**
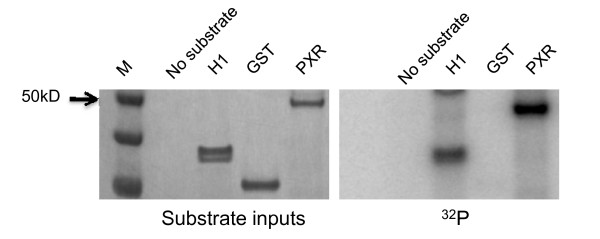
**Cdk5 phosphorylates PXR *in vitro***. Kinase assays were performed as described under Methods. Histone H1 (H1) was the positive and GST the negative control. The amount of substrates loaded to PAGE (substrate inputs) was illustrated by SimplyBlue staining. M, molecular weight marker.

One possible mechanism by which Cdk5 regulates PXR is by directly phosphorylating PXR. All Cdks recognize the same motif for phosphorylation, and Cdk2 [9] and Cdk1 [37] have been shown to phosphorylate PXR. As expected, in an *in vitro *kinase assay, reconstituted complexes of purified Cdk5/p35 directly phosphorylated PXR (Fig. 5), suggesting that Cdk5 can directly phosphorylate hPXR.

### Inhibition of multiple Cdks might contribute to flavonoids-mediated activation of PXR

Since flavonoids have been reported to inhibit multiple Cdks, we investigated the inhibitory effect of flavonoid apigenin on various Cdks. Apigenin inhibited multiple Cdks, including Cdk2, 4, 5, 7, 8, 9 and 11 (Table 1). Since Cdk2 has been previously shown to negatively regulate PXR function [9], these data suggest that inhibition of multiple Cdks might contribute to the activating effect of flavonoids on PXR.

**Table 1:** Apigenin inhibits multiple Cdks.

Kinase	Kd (μM)
Cdk2	9.8

Cdk3	> 30

Cdk4	7.0

Cdk5	3.9

Cdk7	1.7

Cdk8	5.8

Cdk9	2.0

Cdk11	3.0

## Discussion

The widespread use of flavonoids has triggered several studies to investigate the molecular mechanisms of action of these naturally occurring compounds. Flavonoids have been reported to inhibit protein kinases such as Cdks [20] and induce the expression of drug-metabolizing enzymes such as CYPs [31-36]. The stimulatory effect of flavonoids on CYP expression might have significant implication on the pharmacokinetics of drugs co-administered with herbal remedy and potential herbal-drug interactions.

In a cell-based screening approach designed to identify activators of PXR, we identified that flavones luteolin, apigenin and chrysin and isoflavones daidzein, biochanin A, prunetin, and genistein are activators of PXR-mediated *CYP3A4 *gene expression. Genistein and daidzein have been previously reported to activate PXR [35]. In our study, the lack of potent binding of chrysin, luteolin and apigenin to PXR suggests that mechanisms other than direct PXR binding might be responsible for PXR activation by these flavonoids, and the reported inhibitory effect of flavonoids on Cdks (such as Cdk5) led us to investigate the functional relationship between inhibition of Cdk5 and activation of PXR.

We first showed that p35, a critical regulatory protein for Cdk5, is expressed in the human liver carcinoma cell line HepG2. We found an inverse correlation between Cdk5 activity and PXR activity: downregulation of Cdk/p35 signaling activated whereas its upregulation inhibited PXR. In addition, flavonoids restored the Cdk5-mediated downregulation of *CYP3A4 *promoter activity. We further showed that Cdk5/p35 directly phosphorylated PXR.

Cdk5, unlike its regulatory subunit p35, is ubiquitously expressed. The expression of p35 is highest in the nervous system, and has been reported in many non-CNS cells and tissues such as lens epithelia [27], muscle tissues [28] hepatoma cells [25], adipose tissues [29] and male reproductive system [30]. Our discovery that p35 is expressed in HepG2 human liver carcinoma cells expands the list of cells and tissues that are found to express p35. p35 can be cleaved to generate the highly active p25 and we show that calpeptin, a peptide previously reported to inhibit the cleavage of p35 [23,24], highly induced PXR activity and blocked the inhibitory effect of Cdk5 on PXR, supporting that Cdk5 negatively regulates PXR activity.

As with other Cdk inhibitors, the inhibitory effect of flavonoids is not specific to Cdk5, as suggested by inhibition of multiple Cdks by apigenin in the Cdk kinase profiling assay. Cdk2 has been previously shown to negatively regulate PXR function [9]. Our data suggest that flavonoid-mediated activation of PXR is not because of the inhibition of Cdk5 only; inhibition of multiple Cdks, including Cdk2, might contribute to this activation.

Gene expression of *CYP3A4 *is regulated not only by PXR but also by other signaling pathways including other nuclear receptors. These signaling pathways might also cross-talk with each other. Therefore, it is important to investigate the regulation of other signaling pathways and nuclear receptors by flavonoids and the implications in the regulation of gene expression of *CYP3A4 *and other *CYPs*. It is also possible that metabolites of flavonoids may play roles in this complex regulation network. Comprehensively investigating the signaling network regulated by flavonoids and their metabolites will contribute to understanding the roles of flavonoids in potential herbal-drug interactions.

## Conclusions

In conclusion, this is the first report that correlates the effect of flavonoids on regulation of expression of drug-metabolizing enzymes to their inhibitory effect on Cdks, which in turn negatively regulates PXR function. Because of the widespread use of flavonoids by humans as dietary constituents, our discovery may have important implications on the pharmacokinetics of drugs co-administered with herbal remedy and herbal-drug interactions.

## Methods

### Compounds, antibodies, and other materials

Cell culture reagents were obtained from Invitrogen (Carlsbad, CA); compounds and anti-β-actin antibody from Sigma-Aldrich (St. Louis, MO); purified Cdk5-p35 complex and ATP from Millipore (Billerica, MA); purified human PXR protein from Origene Tech (Rockville, MD); [γ-^32^P] ATP from PerkinElmer Life Sciences (Waltham, MA); charcoal/dextran-treated FBS from Hyclone (Logan, UT); Bradford reagent from Bio-Rad (Hercules, CA); and anti-Cdk5 (sc-249) and anti-p35 (sc-821) antibodies from Santa Cruz Biotechnology (Santa Cruz, CA).

### Cell lines, plasmids and transfection

HepG2 liver carcinoma cells, IMR-32 neuroblastoma cells, and HEK 293T kidney epithelial cells were obtained from the American Type Culture Collection (ATCC, Manassas, VA). All cells were maintained at 37°C in a humidified atmosphere containing 5% CO_2_. HepG2 and HEK293T were maintained in modified Eagle's minimal essential medium (MEM) from ATCC with 10% FBS, 2 mM L-glutamine and 100 U/ml penicillin/streptomycin. IMR-32 was maintained in modified Eagle's MEM supplemented with 10% FBS. The Cdk5 expression construct (Cdk5-HA) was provided by Dr. Sander van den Heuvel (Addgene plasmid 1872; Addgene, Cambridge, MA). pCMV-mycP35 was provided by Dr. Li-huei Tsai (Addgene plasmid 1347). FLAG-PXR and *CYP3A4*-luc plasmids were constructed as previously described [9]. Transfections were conducted with FuGENE 6 (Roche Diagnostics), according to manufacturer's instructions.

### PXR transactivation assay

Compounds were added to 384-well plates seeded with cells in a phenol red-free medium containing 5% charcoal/dextran-treated FBS and incubated for 24 h at 37°C before conducting the luciferase assay. The final concentration of DMSO in each well was maintained at 0.1%. DMSO and 2 μM of rifampicin were used as the negative and positive control, respectively. Luciferase activities were detected by using EnVision plate reader (PerkinElmer Life Sciences), as previously reported [9].

### PXR binding assay

The time-resolved fluorescence resonance transfer (TR-FRET) PXR competitive binding assay was performed as described previously [9]. Briefly, the assays were performed in a volume of 20 μl in 384-well solid black plates with 5 nM GST-hPXR ligand-binding domain, 40 nM fluorescent-labeled hPXR agonist (Fluomore PXR Green), 5 nM terbium-labeled anti-GST antibody, and test compound at different concentrations. The reaction mixture was incubated at 25°C for 30 min and then fluorescent emissions of each well were measured at 495 nm and 520 nm, using an excitation filter of 340 nm, a delay time of 100 μs, and an integration time of 200 μs on a PHERAStar plate reader (BMG Labtech, Durham, NC). The FRET ratio was calculated by dividing the emission signal at 520 nm by that at 495 nm. DMSO was used as the negative control (0% relative binding) and 10 μM SR-12813, a human PXR agonist, as the positive control (100% relative binding). The data were expressed as relative binding (%) [relative binding (%) = 100% × (DMSO FRET ratio - Compound FRET ratio)/(DMSO FRET ratio - 10 μM SR12813 FRET ratio)]. Curves were generated by using GraphPad Prism 4.0 (GraphPad Software, La Jolla, CA).

### siRNA knockdown

Endogenous Cdk5 was knocked down by using ON-TARGETplus SMARTpool Cdk5 siRNA (L-003239-00; Thermo Scientific, Chicago, IL). Knockdown efficiency of the target gene was confirmed by Western blotting, as described previously [9]. Briefly, HepG2 cells (2 × 10^5^) were seeded in 6-well plates in serum-free Eagle's MEM. Cells were transfected with 100 pmol siRNA, using Lipofectamine 2000 (Invitrogen). After 6 h, the medium was replaced by Eagle's MEM containing 10% FBS and cells were allowed to grow uninterrupted for 42 h. Cells were then transfected with a total of 1 μg of plasmid DNA by using Fugene 6, cultured for another 24 h, and treated and processed for the luciferase assay or Western blotting.

### In vitro Cdk kinase assay

For the *in vitro *kinase assay, 20 ng of recombinant Cdk5/p35 was used per reaction. Kinase assays were performed in 25 μl reactions, with 1 μg substrate protein PXR, 0.5 μmol/l cold ATP and 5 μCi [γ-^32^P] ATP (6000 Ci/mmol). GST was expressed and purified by using pGEX-4T-1 in *Escherichia coli *BL21 and was used as the negative control. The reaction mixture was incubated at 30°C for 30 min before being electrophoresed by SDS-PAGE. The gel was stained by using SimplyBlue SafeStain (Invitrogen), desiccated by the Labconco gel dryer (Labconco, Kansas City, MO), and exposed overnight to the Storage Phosphor Screen (GE Healthcare). Phosphoimages were obtained by using the Storm scanner (GE Healthcare). *In vitro *Cdks kinase profiling assays were performed by Ambit Biosciences (San Diego, CA) as previously described [38,39].

### Statistical analyses

Results are expressed as the mean ± SD of at least three independent experiments as indicated. The Student's *t*-test for the paired samples was used to determine statistical significance of difference between parameters. Differences were considered significant for *p *< 0.05, 0.01 or 0.001 and non-significant for *p *> 0.05.

## Abbreviations

PXR: pregnane × receptor; CYP: cytochrome P450; Cdk: cyclin-dependent kinase.

## Authors' contributions

HD carried out the transactivation, Western blotting and kinase assays and corresponding data analysis and drafted the manuscript. WL carried out the compound screening and binding assays and corresponding data analysis. JW carried out the construction of plasmids and stable cell line. JW and WL also analyzed the effect of flavonoids and calpeptin on restoring the Cdk5-mediated attenuation of PXR function. TC conceived of and coordinated the design and implementation of the study, and wrote the final manuscript. All authors read and approved the final manuscript.
